# Robust machine learning and ensemble learning approach to predict variation in experimental data for multiple measurements and anomalies

**DOI:** 10.1007/s44211-026-00919-9

**Published:** 2026-04-24

**Authors:** Yuta Sakai, Motosuke Katayama, Hiromasa Kaneko

**Affiliations:** 1 Department of Applied Chemistry, School of Science and Technology, Meiji University, 1-1-1 Higashi-Mita, Tama-ku, Kawasaki, Kanagawa 214-8571 Japan; 2 Nitto Denko Corporation, 1-1-2 Shimohozumi, Ibaraki, Osaka 567-8680 Japan

**Keywords:** Robust machine learning, Ensemble learning, Variability prediction, Repeated measurements, Abnormal values, Outlier robustness, Model selection, Double cross-validation

## Abstract

**Abstract:**

In the field of chemistry, machine learning is widely used to develop desired properties and activities *y* of compounds and products from experimental conditions *x*. As *y* is measured several times, a mathematical model is constructed with *y* as the average value of these measurements, which cannot evaluate the variability of *y*. Therefore, a method was proposed to predict the variability of *y* by creating N sub-datasets with selected *y* values for each sample and constructing multiple models. However, this method reduces prediction accuracy when there are abnormal values due to measurement errors. To address these issues, we proposed a robust method that constructs multiple sub-datasets and selects only the models with the lowest mean absolute error for predictions. Validation on a film thickness and haze (light scattering intensity) dataset showed that the proposed method outperformed conventional approaches, including those that remove anomalies in advance, in predicting both the mean and variation of *y*. The proposed method could improve accuracy in datasets with multiple values of *y* and containing abnormal values without removing samples.

**Graphical abstract:**

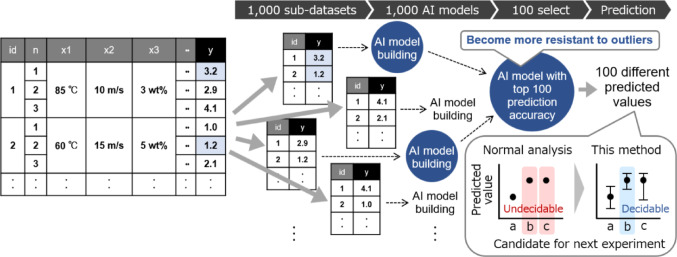

## Introduction

In the field of chemistry, machine learning has been used to design compounds and products with desired activities and properties. In general, a mathematical model *y* = *f*(*x*) is constructed using information such as previously obtained experimental conditions as the explanatory variable *x* and the desired properties as the objective variable *y*. Predicted *y* are obtained by inputting *x*, such as new experimental conditions into this model, and experiments are conducted under experimental conditions with the desired predicted *y*.

Typically, the properties of a product or compound are measured several times. In machine learning analysis, the average of these measurements is often used as *y*. For example, a model was constructed to predict the rate of bone formation of artificial bone from features calculated from scanning electron microscopy images of artificial bone with feature engineering and latent variables obtained by constructing an auto-encoder [1], the average of multiple measurements of bone formation rate was used as *y*. Other examples, two models were proposed to design the chemical structure of a novel hypercholesterolemia drug with both high inhibitory activity and high hepatic selectivity for 3-hydroxyl-3-methyl glutaryl coenzyme A reductase inhibitory activity and the organic anion transporting polypeptide 1B1 affinity [2], duplicate samples in the IC_50_ dataset collected from the ChEMBL database were IC_50_ values were calculated by averaging. However, if the average value is *y*, it is not possible to predict how *y* will vary for each product, as the predicted result obtained is also the average value of *y*. Another problem is that when the predicted results of variability are obtained, it is not possible to evaluate the variability of the predicted values in relation to the variability of the actual measured values. To solve these problems, a method was proposed to predict the variability of bone formation rate of artificial bone [3]. In this method, to predict the variability from a dataset with multiple measurements of bone formation rate, 100 sub-datasets were created by randomly extracting the measured values of bone formation rate from the dataset for each sample. By constructing a sub-model with each sub-dataset, 100 sub-models were obtained. By inputting *x* for a sample into these sub-models, 100 *y* values were predicted, which allowed us to predict the variability of *y*. Furthermore, by assuming that the variation in bone formation rate follows a normal distribution and using the Jensen-Shannon divergence (JSD), which quantifies the distance between distributions, as an evaluation metric. The prediction error of the sample variability can be obtained by calculating the JSD of the distribution of the measured and predicted values of the sample. However, due to malfunction of the measuring equipment or human error, some measurement results may contain abnormal values that are different from other values. In such cases, the variation of *y* is far from the original variation of the product due to the influence of the abnormal values. If a machine learning model is built using *y* containing such abnormal values, the prediction accuracy can be lower than that of a model built without abnormal values.

Therefore, this study proposes a variability prediction model that is robust to outliers or abnormal values in *y* by extending a previously proposed variability-prediction framework [3] with an additional model-selection step based on predictive accuracy. This method is validated on a real product dataset. The dataset contains one *y* without anomalies and another *y* with anomalies, and it was confirmed that the proposed method is more accurate than the conventional method in both cases. In the dataset containing anomalies, the accuracy was equal to or better than the model constructed by removing the anomalies, confirming that the proposed method is robust to the dataset with anomalies in *y*.

## Method

### **Dataset**

**Fig. 1:**
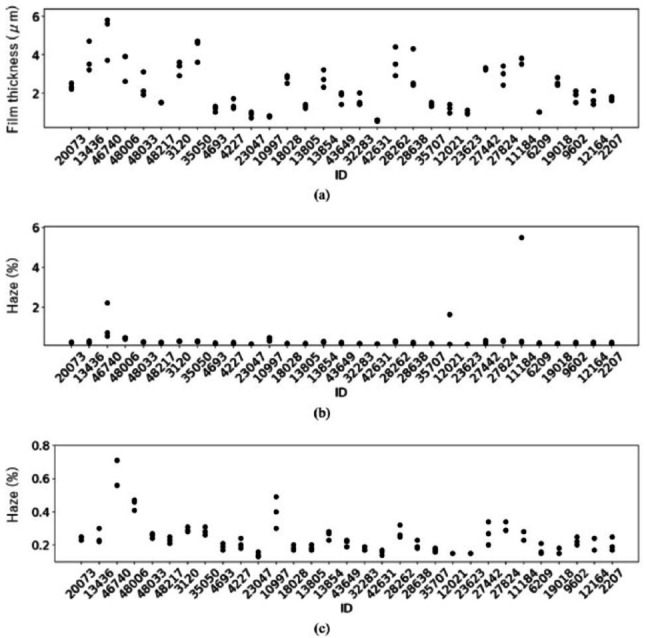
Measured objective variables for each sample. **a** Film thickness, **b** Haze with abnormal values, **c** Haze without abnormal values

In this study, a dataset for a specific product provided by Nitto Denko Corporation was used, and the dataset is summarized in Table 1. The experimental samples consisted of films coated onto glass substrates. As the explanatory variables (x), seven variables related to product manufacturing, such as coating solution concentration, were considered, while the response variables (y) were film thickness and haze. Film thickness was measured in micrometers (µm) using a white light interferometric thickness gauge, and haze was measured in percentage (%) using a haze meter based on ISO 14,782. For a product with 31 samples, each of these *y* was measured three times. Figure 1. shows the measured values of film thickness and haze that are *y* for each sample. There are no significant abnormal values for film thickness. On the other hand, the haze has three IDs (11184, 46740 and 12021) with values larger than 1 for each sample. These measured values are clearly abnormal compared to the other measured values of the same sample, and since they were suspected to be caused by errors during sample preparation, they were not regarded as mere statistical outliers but were instead identified and treated as abnormal values.

**Table 1:** Summary of the dataset used in this study

ID	x1	x2	x3	x4	x5	x6	x7	y1; Film thickness (μm)	y2; Haze (%)
20073_1	50	130	1	15	10.0	80	2	2.50	0.25
20073_2	50	130	1	15	10.0	80	2	2.20	0.25
20073_3	50	130	1	15	10.0	80	2	2.30	0.23
13436_1	50	60	15	60	10.0	120	1	3.50	0.23
13436_2	50	60	15	60	10.0	120	1	4.70	0.30
13436_3	50	60	15	60	10.0	120	1	3.20	0.22
46740_1	100	150	0	1	2.0	120	1	5.60	0.56
46740_2	100	150	0	1	2.0	120	1	5.80	0.71
46740_3	100	150	0	1	2.0	120	1	3.70	2.21
48006_1	100	150	0	1	1.0	150	1	2.60	0.41
48006_2	100	150	0	1	1.0	150	1	3.90	0.46
48006_3	100	150	0	1	1.0	150	1	3.90	0.47
48033_1	100	150	0	1	10.0	80	2	3.10	0.27
48033_2	100	150	0	1	10.0	80	2	2.10	0.26
48033_3	100	150	0	1	10.0	80	2	1.90	0.24
48217_1	100	150	1	15	2.0	80	2	1.50	0.25
48217_2	100	150	1	15	2.0	80	2	1.50	0.23
48217_3	100	150	1	15	2.0	80	2	1.50	0.21
3120_1	25	90	15	30	1.0	80	1	2.90	0.31
3120_2	25	90	15	30	1.0	80	1	3.60	0.29
3120_3	25	90	15	30	1.0	80	1	3.40	0.28
35050_1	75	150	15	1	1.5	100	1	3.60	0.26
35050_2	75	150	15	1	1.5	100	1	4.70	0.28
35050_3	75	150	15	1	1.5	100	1	4.60	0.31
4693_1	25	90	1	15	1.5	120	2	1.00	0.19
4693_2	25	90	1	15	1.5	120	2	1.30	0.21
4693_3	25	90	1	15	1.5	120	2	1.20	0.17
4227_1	25	90	5	15	5.0	100	2	1.30	0.20
4227_2	25	90	5	15	5.0	100	2	1.70	0.24
4227_3	25	90	5	15	5.0	100	2	1.20	0.18
23047_1	50	180	0	1	1.0	150	2	0.70	0.14
23047_2	50	180	0	1	1.0	150	2	0.90	0.16
23047_3	50	180	0	1	1.0	150	2	1.00	0.13
10997_1	25	180	0	30	10.0	120	2	0.80	0.30
10997_2	25	180	0	30	10.0	120	2	0.76	0.49
10997_3	25	180	0	30	10.0	120	2	0.81	0.40
18028_1	50	130	0	30	5.0	120	1	2.80	0.20
18028_2	50	130	0	30	5.0	120	1	2.50	0.18
18028_3	50	130	0	30	5.0	120	1	2.90	0.17
13805_1	50	60	5	15	1.0	120	2	1.40	0.20
13805_2	50	60	5	15	1.0	120	2	1.20	0.18
13805_3	50	60	5	15	1.0	120	2	1.30	0.17
13854_1	50	60	5	30	1.5	150	1	3.20	0.28
13854_2	50	60	5	30	1.5	150	1	2.70	0.27
13854_3	50	60	5	30	1.5	150	1	2.30	0.23
43649_1	100	130	0	60	1.5	80	2	1.40	0.22
43649_2	100	130	0	60	1.5	80	2	1.90	0.19
43649_3	100	130	0	60	1.5	80	2	2.00	0.23
32283_1	75	130	1	60	1.0	100	2	2.00	0.19
32283_2	75	130	1	60	1.0	100	2	1.50	0.17
32283_3	75	130	1	60	1.0	100	2	1.40	0.17
42631_1	100	90	5	15	5.0	150	2	0.60	0.14
42631_2	100	90	5	15	5.0	150	2	0.55	0.16
42631_3	100	90	5	15	5.0	150	2	0.54	0.17
28262_1	75	90	0	30	2.0	150	1	2.90	0.25
28262_2	75	90	0	30	2.0	150	1	3.50	0.26
28262_3	75	90	0	30	2.0	150	1	4.40	0.32
28638_1	75	90	5	60	10.0	150	1	2.50	0.18
28638_2	75	90	5	60	10.0	150	1	4.30	0.23
28638_3	75	90	5	60	10.0	150	1	2.40	0.19
35707_1	75	150	15	1	5.0	100	2	1.40	0.17
35707_2	75	150	15	1	5.0	100	2	1.30	0.18
35707_3	75	150	15	1	5.0	100	2	1.50	0.16
12021_1	25	180	15	1	2.0	120	2	1.40	1.65
12021_2	25	180	15	1	2.0	120	2	1.20	0.15
12021_3	25	180	15	1	2.0	120	2	0.95	0.15
23623_1	50	180	15	30	2.0	150	2	1.10	0.15
23623_2	50	180	15	30	2.0	150	2	0.92	0.15
23623_3	50	180	15	30	2.0	150	2	0.97	0.15
27442_1	75	60	15	30	1.0	100	1	3.20	0.27
27442_2	75	60	15	30	1.0	100	1	3.30	0.34
27442_3	75	60	15	30	1.0	100	1	3.30	0.20
27824_1	75	60	1	60	5.0	80	1	2.40	0.29
27824_2	75	60	1	60	5.0	80	1	3.00	0.34
27824_3	75	60	1	60	5.0	80	1	3.40	0.29
11184_1	25	180	1	60	5.0	80	1	3.80	5.50
11184_2	25	180	1	60	5.0	80	1	3.50	0.23
11184_3	25	180	1	60	5.0	80	1	3.80	0.28
6209_1	25	130	5	60	1.5	80	2	1.00	0.16
6209_2	25	130	5	60	1.5	80	2	1.00	0.15
6209_3	25	130	5	60	1.5	80	2	1.00	0.21
19018_1	50	130	5	60	2.0	100	1	2.80	0.18
19018_2	50	130	5	60	2.0	100	1	2.50	0.15
19018_3	50	130	5	60	2.0	100	1	2.40	0.15
9602_1	100	60	0	1	1.0	80	2	1.50	0.22
9602_2	100	60	0	1	1.0	80	2	1.90	0.20
9602_3	100	60	0	1	1.0	80	2	2.10	0.25
12164_1	100	180	0	1	1.0	100	2	2.10	0.24
12164_2	100	180	0	1	1.0	100	2	1.40	0.24
12164_3	100	180	0	1	1.0	100	2	1.60	0.17
2207_1	25	150	1	60	1.0	150	1	1.60	0.25
2207_2	25	150	1	60	1.0	150	1	1.80	0.17
2207_3	25	150	1	60	1.0	150	1	1.70	0.19

In this study, *x* represents fixed experimental setting values for each sample, and therefore variability in *x* is not considered. The target of this study is the variability in *y* obtained from repeated measurements under fixed *x*.

### **Proposed method**

**Fig. 2:**
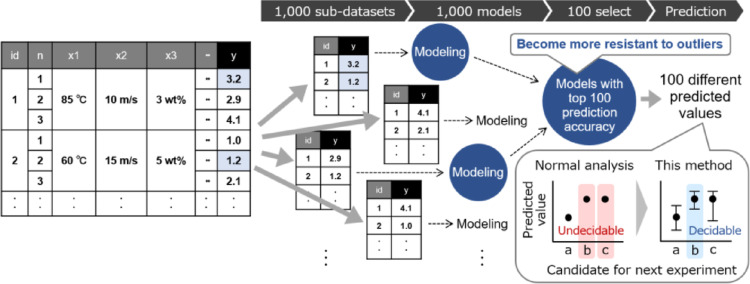
Concept of the proposed method

This study proposes a novel approach for constructing and selecting multiple prediction models to ensure robust prediction accuracy while decreasing the influence of abnormal values. Figure 2 shows the concept of the proposed method. First, *M* sub-datasets are generated from the original dataset. Sub-datasets are created by randomly selecting a set of actual measurements of *y* for all samples. This ensures that *x* and *y* are 1:1 in all sub-datasets. When the number of original samples (the number of ids in Fig. 2) is K, the number of samples in each sub-dataset is *K*.

Next, for each of the *M* sub-datasets, an individual mathematical model *y* = *f*(*x*) is constructed using machine learning. As a result, *M* different models are created, a regression method and its hyperparameters of each model are optimized with a corresponding sub-dataset. To evaluate the predictive accuracy of these models, mean absolute error (MAE) of each model is calculated using double cross-validation (DCV) [4]. The top *N* models with the lowest MAE are selected from the *M* models. In other words, *N* models with high prediction accuracy are selected. This selection ensures that only the most reliable models, which are less influenced by abnormal values, are used in the final prediction process.

Then, the selected *N* models are used to predict *y* for *x* of a sample. These predictions are confirmed and evaluated in three different ways. First, all measured values and all predicted values for each sample are plotted on a scatterplot and evaluated by comparing the two distributions. Second, the mean of measured values and the mean of predicted values are plotted on the scatter diagram. Finally, rectangles were added to the plots, where the horizontal length represents the range of the measured values and the vertical length represents the range of the predicted values for each sample. A square-like rectangle indicates similar magnitudes of measured and predicted variability, whereas a horizontally elongated rectangle indicates that the predicted variability is narrower than the measured variability. Because abnormal measurements can artificially broaden the measured range, these rectangles are used as qualitative visual aids and are interpreted together with the scatter plots of all measured and predicted values and the numerical metrics. A better prediction of variation is obtained when the rectangle is located near the diagonal line.

Although no strict theoretical criterion is currently available for determining *N*, *N* should be sufficiently large to ensure stable and reproducible prediction. In this study, *N* = 100 was considered large enough for this purpose. Since *N* models are selected from *M* candidate models, *M* should be set sufficiently larger than *N*. From an empirical viewpoint, setting *M* to approximately ten times *N* is reasonable; therefore, *M* = 1000 and *N* = 100 were used in this study.

## Results and discussion

### **Comparison of models**

Before presenting the comparison results, the compared methods and the comparison settings are briefly summarized below. The following three methods were used in this study.

Method (A): *M* = 100, *N* = 100, which corresponds to a baseline method based on the previously published framework in reference [3].

Method (B): Remove abnormal values in advance. *M* = 100, *N* = 100.

Method (C): *M* = 1000, *N* = 100.

The coefficient of determination (*r*^2^), calculated from the average of the measured values and the average of the predicted values, was used for both film thickness and haze. For haze only, *r*^2^_drop_ was additionally calculated from the average of the measured values without abnormal values and the average of the predicted values, which was used as metrics to compare the models in Method (A) to (C).

In the present dataset, the number of samples obtained under the same experimental conditions was limited for each condition. Therefore, the actual variation of *y* under each condition could not be accurately quantified, which made rigorous quantitative evaluation of the predicted variation difficult. For this reason, the variation was mainly assessed qualitatively in this study. A more rigorous quantitative evaluation of the predicted variation will require datasets with a larger number of repeated measurements under identical experimental conditions.

In Method (A) and (B), ordinary least squares (OLS) [5], partial least squares regression (PLS) [6], ridge regression (RR) [7], least absolute shrinkage and selection operator (LASSO) [7], elastic net (EN) [7] and linear support vector regression (LSVR) [8] were used as linear methods and Gaussian process regression (GPR) [9], nonlinear support vector regression (NLSVR) [8], decision tree (DT) [10], random forest (RF) [11], gradient boosting decision tree (GBDT) [12], extreme gradient boosting (XGB) [13], light gradient boosting model (LGBM) [13], Gaussian mixture regression (GMR) [14] and variational Bayesian Gaussian mixture regression (VBGMR) [15] as non-linear methods for constructing models in each sub-dataset, and each model was evaluated using DCV (inner fold: 5, outer fold: 10), with the most accurate the method with the method with the lowest MAE was considered optimal for that sub-dataset. Due to the large number of sub-datasets in Method (C), models were constructed for OLS, PLS, RR, Lasso, NLSVM, DT, GP, GBDT, XGB and LGBM, which are computationally less expensive. Multiple linear and nonlinear regression methods were compared because the most suitable model structure was not known a priori, and allowing model selection for each sub-dataset reduced dependence on any single regression method.

### **Model construction**

**Fig. 3:**
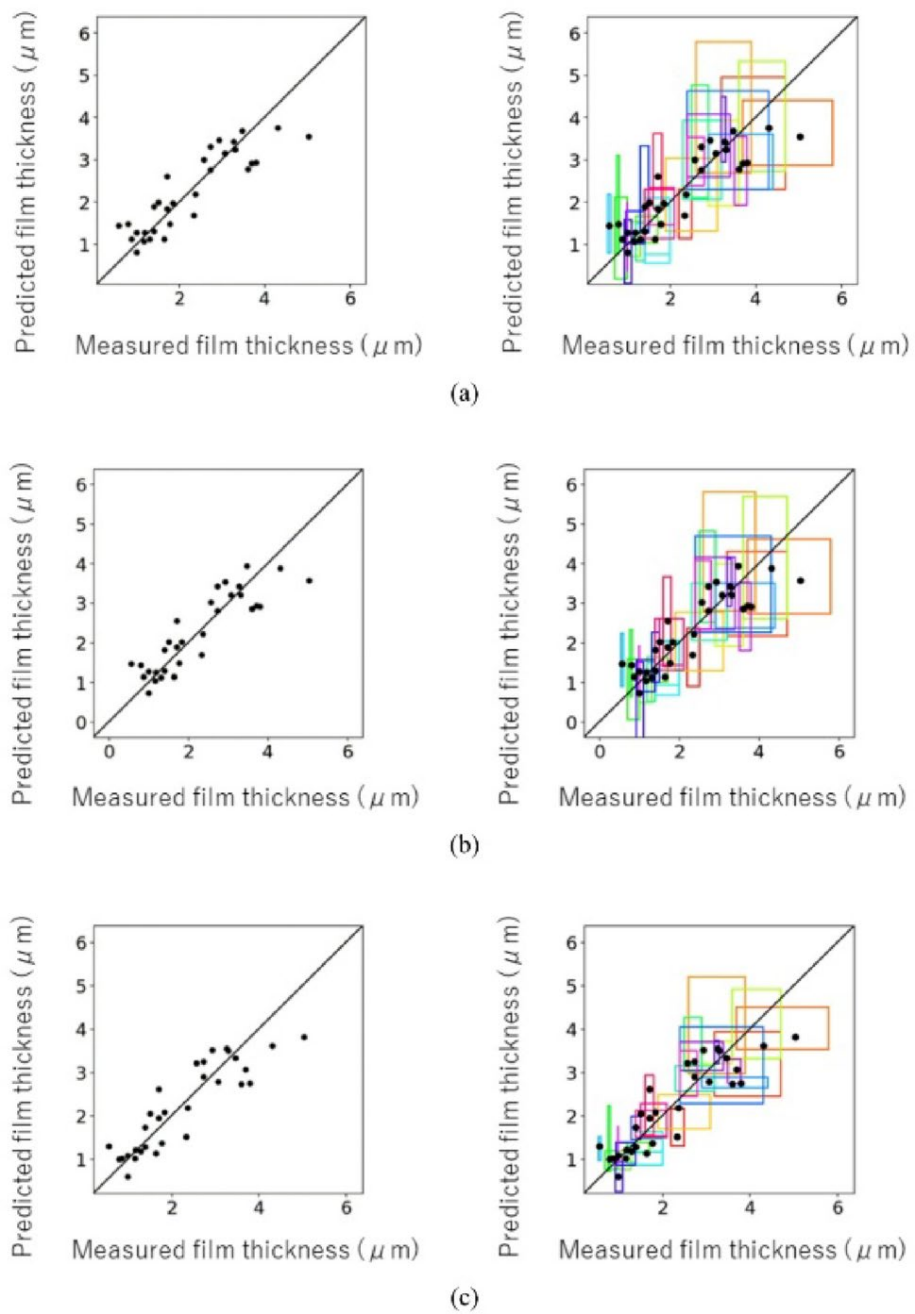
Scatter plots of each average of the predicted and measured film thickness values with rectangles showing the dispersion. **a** Method (A), **b** Method (B), **c** Method (C)

First, film thickness was investigated. Since no abnormal values were identified for film thickness, *r*^2^_drop_ was not defined for film thickness, the comparison was based on *r*^2^ in Table 2, and Method (B) should be interpreted only as a reference condition. Method (C) showed the highest *r*^2^ among the three methods. Figure 3 shows scatter plots of the mean of the measured values and the mean of the predicted values, together with rectangles representing the ranges of the measured and predicted values for each sample. The mean values were predicted reasonably well by all methods, whereas Method (C) placed the samples with relatively high film thickness closer to the diagonal line than the other methods, which was consistent with the highest *r*^2^.

**Table 2:** Methods and metrics

Method	*r*^2^ for film thickness	*r*^2^ for haze	*r*^2^_drop_ for haze
A	0.778	− 0.012	0.290
B	0.770	− 0.024	0.461
C	0.783	− 0.008	0.464

**Fig. 4:**
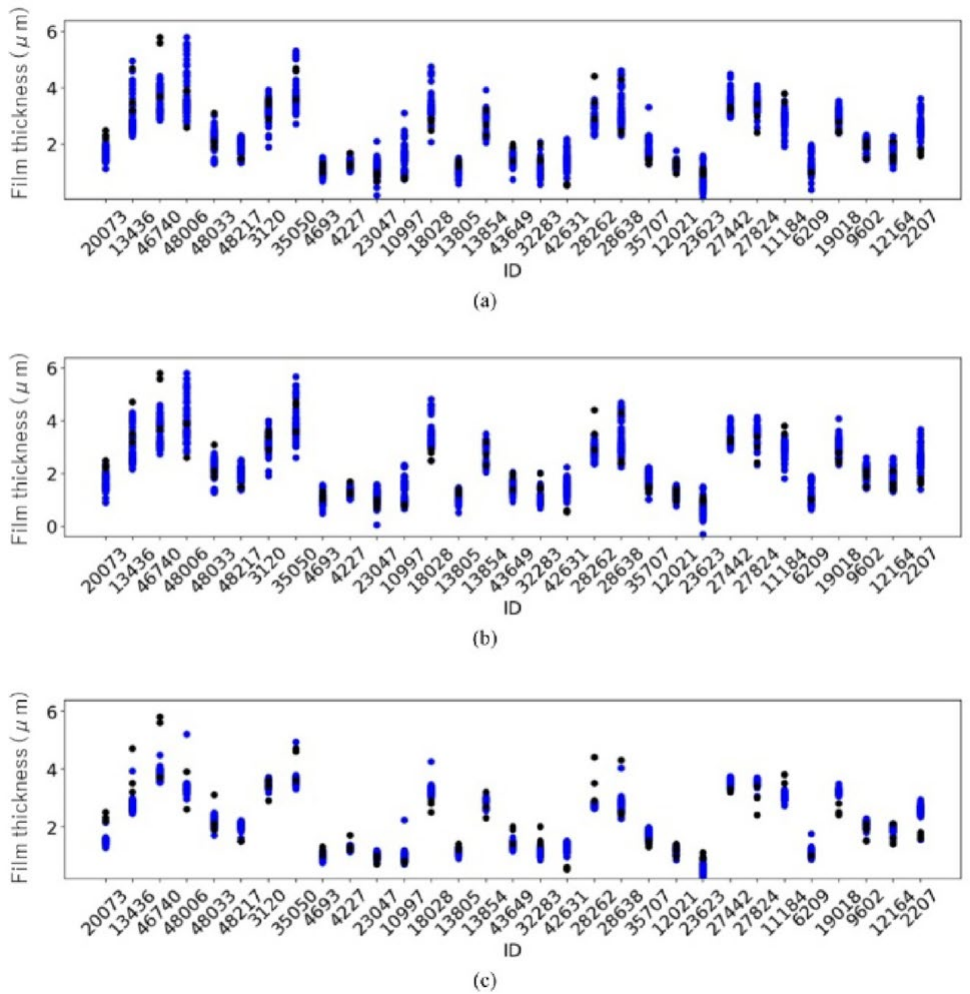
Measured and predicted film thickness values for each sample. Black points indicate measured values and blue points indicate predicted values. All 100 predicted values are plotted in order to visualize the full distribution of the predictions for each sample, including the spread and the presence of unusually large or small predicted values. **a** Method (A), **b** Method (B), **c** Method (C)

Regarding the rectangles, the important point is not whether each rectangle is perfectly square, but whether the horizontal range of the predicted values is comparable to the vertical range of the measured values and whether the rectangle is located near the diagonal line. From this viewpoint, Method (C) reduced samples showing excessively broad predicted ranges or clear deviations from the measured ranges. This tendency is also supported by Fig. 4, in which Methods (A) and (B) spread predicted values over a wider range, whereas Method (C) produced predicted values mostly near the corresponding measured values. These results suggest that selecting low-MAE models from a large number of candidate models suppresses sub-datasets that lead to unrealistic variability.

**Fig. 5:**
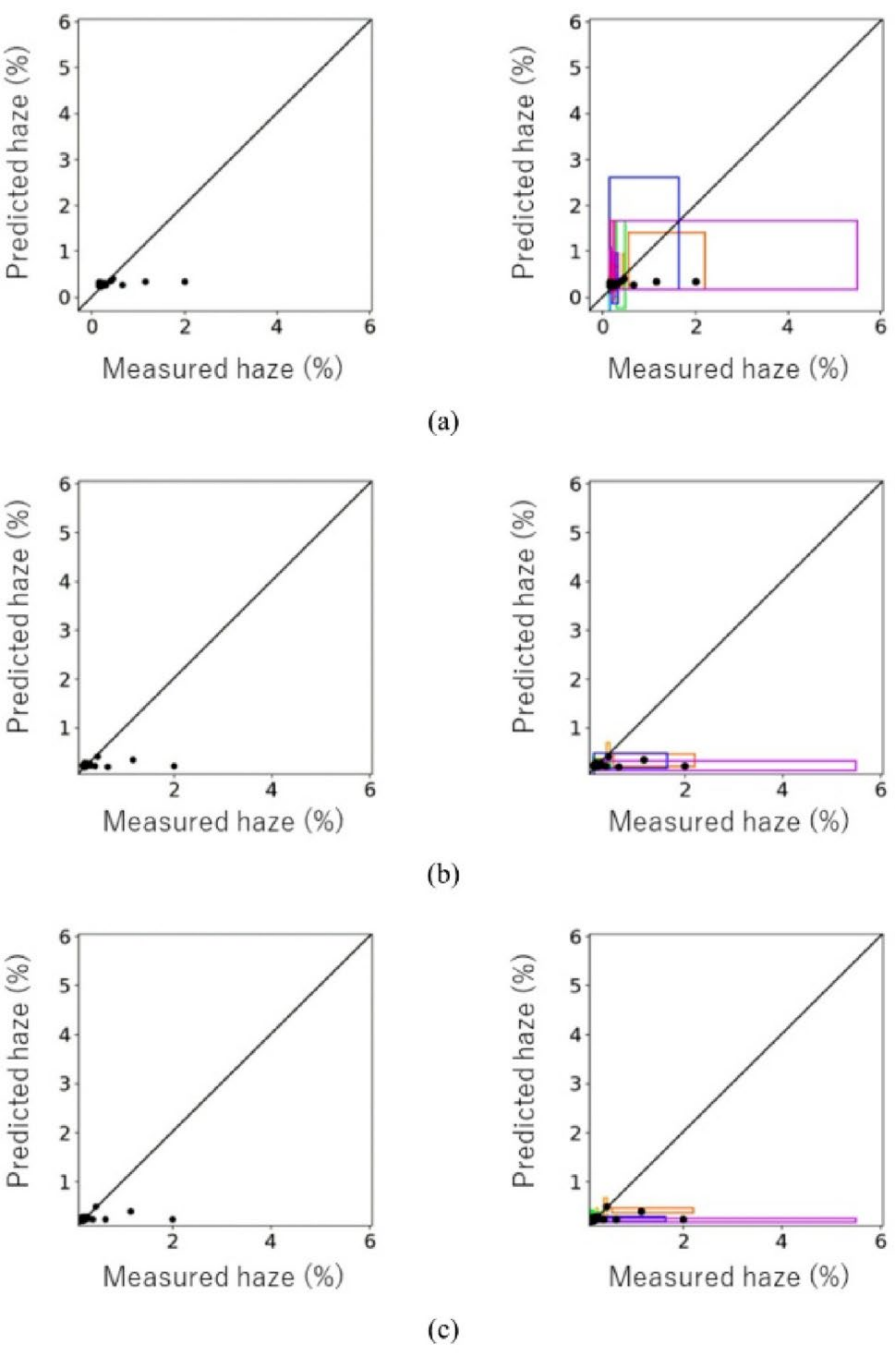
Scatter plots of each average of the predicted haze values with abnormal values and measured haze values with rectangles showing dispersion. **a** Method (A), **b** Method (B), **c** Method (C)

**Fig. 6:**
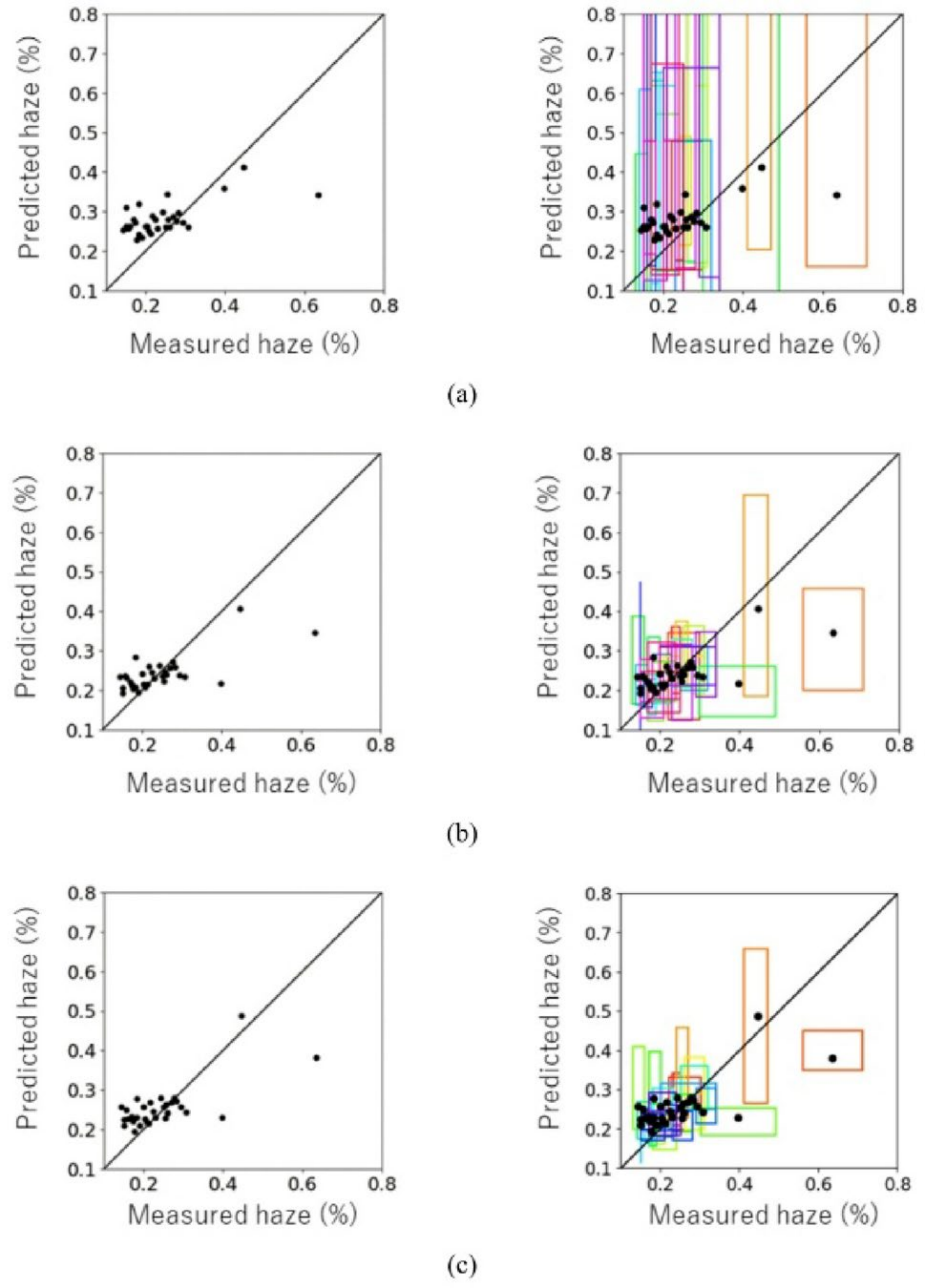
Scatter plots of each average of the predicted haze values without abnormal values and measured haze values with rectangles showing the dispersion. **a** Method (A), **b** Method (B), **c** Method (C)

Next, haze, which contained three abnormal values, was investigated. For haze, the ordinary *r*^2^ values were negative because they were strongly affected by the abnormal values. Therefore, direct comparison among the methods based on these *r*^2^ values is not appropriate, and the methods were mainly compared using *r*^2^_drop_, in which the abnormal values were excluded. As shown in Table 2, *r*^2^_drop_ was higher for Methods (B) and (C) than for Method (A), indicating that the influence of the abnormal values was reduced in Methods (B) and (C). Figures 5 and 6 show the same type of scatter plots as in Fig. 3. The mean values indicate that Methods (B) and (C) predicted haze more accurately than Method (A), especially in the lower range.

**Fig. 7:**
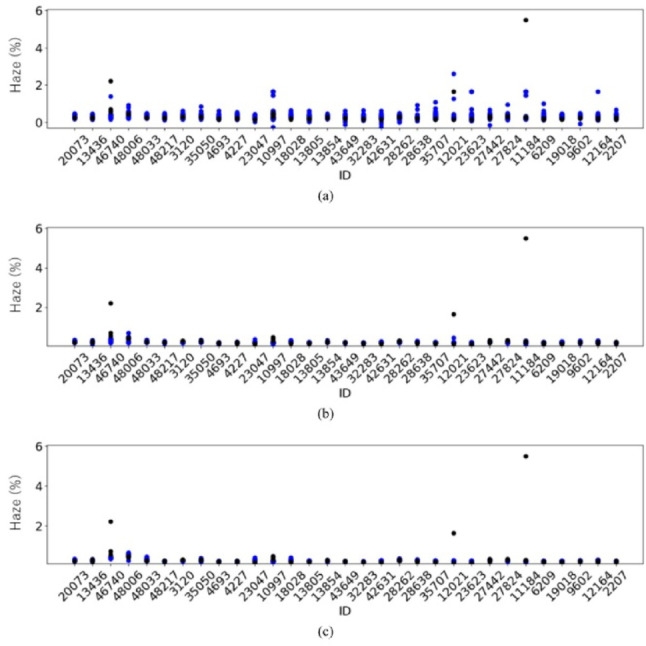
Measured and predicted film thickness values with abnormal values for each sample. Black points indicate measured values and blue points indicate predicted values. All 100 predicted values are plotted in order to visualize the full distribution of the predictions for each sample, including the spread and the presence of unusually large or small predicted values. **a** Method (A), **b** Method (B), **c** Method (C)

**Fig. 8:**
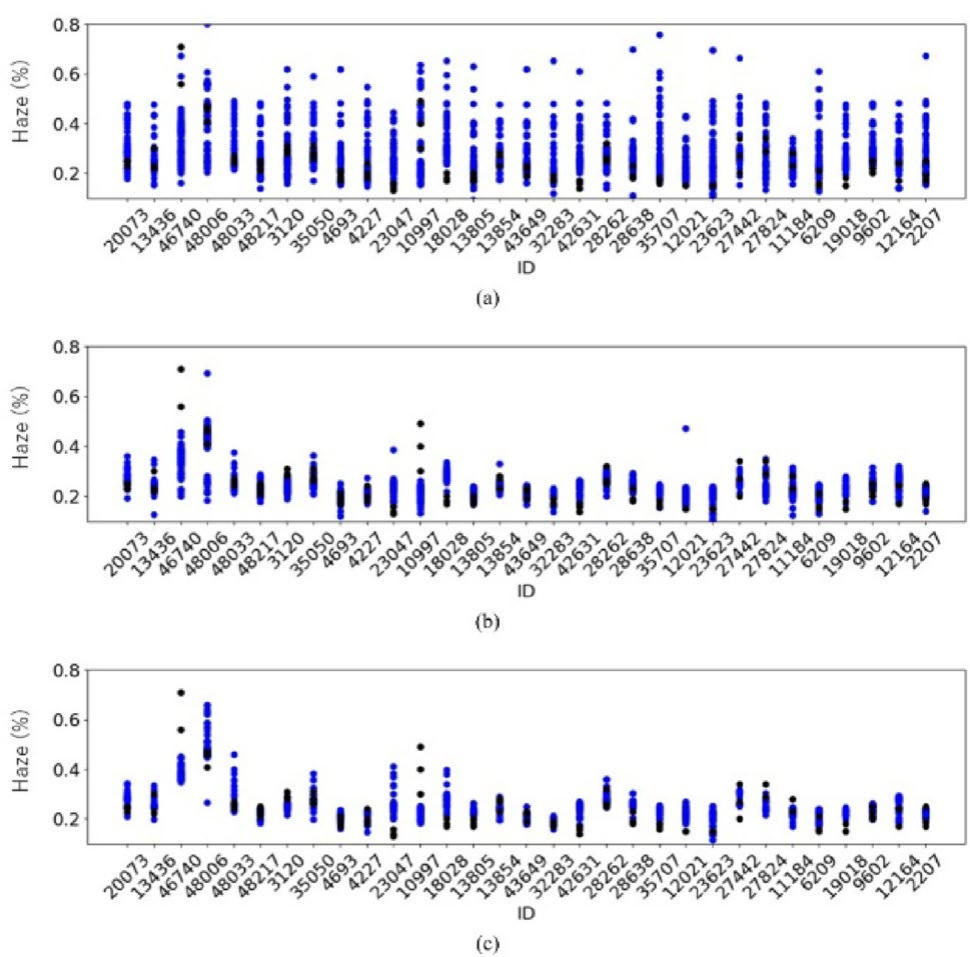
Measured and predicted haze values without abnormal values for each sample. Black points indicate measured values and blue points indicate predicted values. All 100 predicted values are plotted in order to visualize the full distribution of the predictions for each sample, including the spread and the presence of unusually large or small predicted values. (a) Method (A), (b) Method (B), (c) Method (C)

In terms of the rectangles, they should be interpreted as a supplementary visualization of the agreement between the measured and predicted ranges, rather than as a criterion based only on how close they are to squares. Method (A) frequently produced rectangles shifted toward larger predicted values and with wider horizontal ranges than the measured ranges, indicating overestimation of variability due to the abnormal values. In contrast, although some rectangles in Method (C) were still horizontally elongated, their horizontal ranges were generally narrower and their positions were closer to the measured ranges than those in Method (A). This interpretation is consistent with Figs. 7 and 8, where Method (C) suppressed excessively large predicted values compared with Method (A). Therefore, Method (C) achieved a better overall balance between accurate prediction of the mean values and suppression of unrealistic spread caused by abnormal values by selecting only low-MAE models constructed with sub-datasets without abnormal values from a large number of candidate models.

In this study, one *y* had no abnormal values and the other contained three abnormal values. In both cases, Method (C) outperformed Method (A), which indicates that selecting highly accurate models from many candidate models is effective for improving robustness. In the dataset containing abnormal values, Method (C) also achieved performance comparable to or better than Method (B), where the abnormal values were removed in advance. These results support the robustness of the proposed method for datasets with multiple measurements of *y*, even when abnormal values are included.

## Conclusions

In this study, in a dataset where each sample has multiple values of *y*, the proposed method was to build a model with *M* sub-datasets each created by randomly selecting *y* values for each sample and selecting the top *N* models with the highest accuracy. The proposed method was confirmed to be more accurate than the method with *N* sub-datasets and models. It was also confirmed that the model was robust to datasets with anomalies in *y* and was as accurate as or more accurate than the model constructed by removing the anomalies. For the proposed method, *N* should be sufficiently large to ensure stable prediction, while *M* should be set sufficiently larger than *N* so that enough candidate models are generated before selection. From an empirical viewpoint, setting *M* to approximately ten times *N* is a reasonable practical choice. The proposed method can improve accuracy in datasets with multiple values of *y* and containing abnormal values without removing samples.

## Data Availability

All data supporting the fi ndings of this study are available within the article. The dataset used in this study is provided in Table 1.
